# A Look at the Spatial Confining Effect on the Molecular Electrostatic Potential (MEP)—A Case Study of the HF and BrCN Molecules

**DOI:** 10.3390/molecules26195924

**Published:** 2021-09-29

**Authors:** Paweł Lipkowski, Justyna Kozłowska, Wojciech Bartkowiak

**Affiliations:** Department of Physical and Quantum Chemistry, Faculty of Chemistry, Wrocław University of Science and Technology, Wyb. Wyspiańskiego 27, PL-50370 Wrocław, Poland; pawel.lipkowski@pwr.edu.pl (P.L.); justyna.kozlowska@pwr.edu.pl (J.K.)

**Keywords:** molecular electric potentials, spatial confinement, intermolecular interactions

## Abstract

In this theoretical study, we report on the molecular electrostatic potential (MEP) of titled molecules confined by repulsive potentials of cylindrical symmetry mimicking a topology. Our calculations show that the spatial restriction significantly changes the picture of the MEP of molecules in a quantitative and qualitative sense. In particular, the drastic changes in the MEP as a function of the strength of spatial confinement are observed for the BrCN molecule. This preliminary study is the first step in the investigation of the behavior of the MEP of molecular systems under orbital compression.

## 1. Introduction

Incorporation of the molecular electrostatic potential (MEP) concept into the chemistry and molecular physics community is owed to the pioneering works of Scrocco and Tomasi in the early 1970s [1]. Since then, the MEP has advanced to the position of an important tool of computational chemistry in elucidating various properties of atoms, molecules and materials [2,3,4,5]. It results from the ease of interpretation of the MEP in terms of classical electrostatics: the initial tendency of an approaching positive point charge is to go to those parts of the molecule where the MEP is most negative. In addition, the computation of molecular electrostatic potential from the wave function is not a very demanding task; thus, it is feasible to obtain its values at various points around the molecule and represent the MEP as a three- or two-dimensional isopotential map or, by color, on the molecular envelope. Therefore, it is hardly surprising that the introduction of the MEP was an important step toward the domestication of quantum chemistry and making it more accessible to chemists and other experimentalists. The molecular electrostatic potential created in the space around a molecule by its nuclei and electrons is defined rigorously by the following equation (in terms of atomic units) [1,2,3,4,5]:

(1) $$V\left(r\right)=\underset{A}{\sum }\frac{Z_{A}}{\left|R_{A}-r\right|}-\int^{\;}\frac{\rho \left(r^{,}\right)dr^{,}}{\left|r^{,}-r\right|}$$

where *Z_A_* denotes the charge on nucleus A, located at a distance *R_A_*, and *ρ*(*r*′) is the molecule’s electronic density. In contrast to *ρ*(*r*), which represents solely the electronic density at the point *r*, MEP involves contributions from each nucleus and electrons in the entire molecule, thus providing a total electrostatic picture. The first term of Equation (1) brings in the positive contribution of the nuclei, while the second one represents the negative effect of the electrons. Hence, the sign of *V*(*r*) in any particular region depends on whether the nuclear or the electronic term is dominant there. It is worth mentioning that MEP belongs to the quantities that are observable physically, and thus, it can be derived directly from the wave function or experimental electron densities available from, e.g., X-ray diffraction [6]. Typical applications of MEP have expanded from primarily a focus on sites for electrophilic and nucleophilic attack to the interpretation of molecular electronic structure, structure–activity relationships or the theory of solvent effects [2,3,4,5,7,8,9,10], just to name a few. One of the common applications of the molecular electrostatic potential is also the study of long-range interactions [11]. An important type of such interactions, in the context of which the knowledge of MEP has been proven very useful, is the σ-hole bonding. Initially, the σ-hole concept was introduced to resolve the enigma of halogen bonding (XB) [5,7,8,9,10,12,13,14,15]. The explanation of this phenomenon is based on the anisotropy of the electron density distribution around the halogen atom. As it has been demonstrated, the distribution of electron density around the X atom adopts an ellipsoidal form with the longer radius perpendicular to the direction of the R-X bond. This leads to the formation of the electron-deficient region along the R-X direction, which consequently exhibits an excess of positive charge (the so-called σ-hole). The interactions involving σ-holes are highly directional, due to the localization of the electron acceptor site (σ-hole) on the extension of the covalent bond. Driving the molecular organization in the space, σ-hole interactions can control the properties of supramolecular entities and materials through the structure–properties relationship, making them of great importance in the fields of crystal engineering, supramolecular chemistry, nanotechnology and material science. However, it should be noticed that the prominent role of the σ-hole in the context of formation of the halogen bond is also criticized [16].

Recent years have seen a significant strengthening of studies concerning the description of noncovalent complexes exposed to high pressure or embedded in confining environments (see Refs. [17,18] and the references cited therein). In particular, the two-dimensional harmonic oscillator potential was applied in order to render the impact of orbital compression on the analyzed molecular complexes. The energetic analysis performed for the confined H-bonded complexes has shown a different trend in the interaction energy changes [17]. Recently, the interaction energy and bond lengths of FHF-(bifluoride) with increasing confinement were investigated [19]. A similar theoretical description concerning the various properties of the model halogen bonded FCl–CNF complex upon the spatial confinement was also performed [18]. These investigations and observations open up the possibility of constructing molecular systems with entirely new properties, mostly determined by size effects (e.g., endohedral complexes, inclusion compounds or low-dimensional semiconductor structures). Generally, in order to glean a fundamental understanding, in the context of quantitative and qualitative results, of the nature of spatially confined molecular complexes energetics, intermolecular distances or vibration properties are considered, which requires performing high level ab initio molecular orbital calculations. Hence, the search for the simplest solutions and models—for example, based on the analysis of MEP—is an important and necessary challenge.

The main motivation of this work was to examine the spatial confinement effect on the MEP of the titled compounds. This preliminary study is the first step in the investigations of the behavior of the MEP of molecular systems under orbital compression. This work is a continuation of our investigations in this field devoted to theoretical descriptions of the electrical properties (in particular, linear and nonlinear) of atomic and molecular systems (see, e.g., Refs. [17,18,20,21,22,23]).

## 2. Results and Discussion

**Figure 1:**
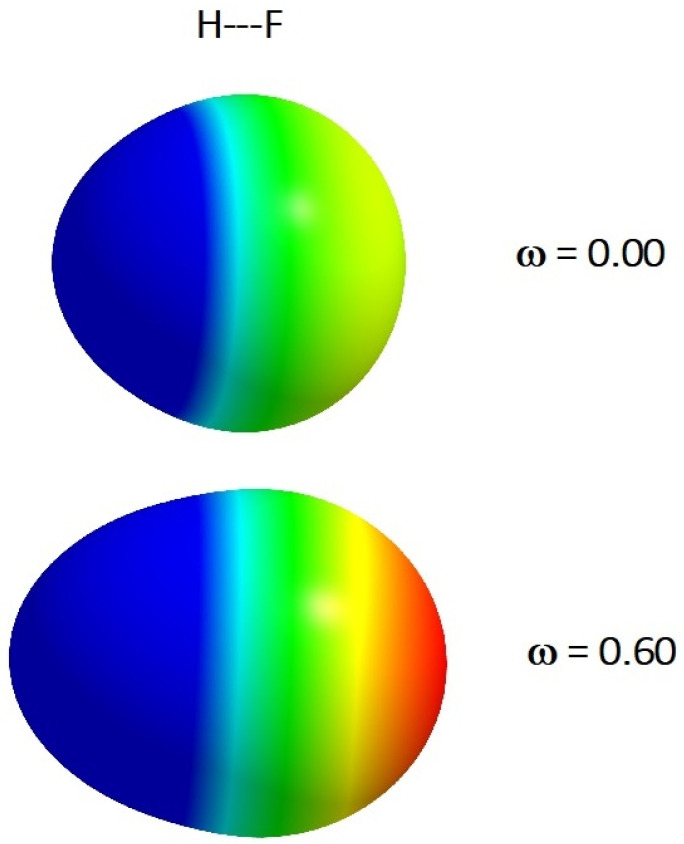
The *ω*B97X/aug-cc-pVTZ calculated MEP on the 0.001 a.u. isodensity surface of HF molecule (for the free molecule as well as for the molecule upon confinement). The meaning of the colors is explained in the text.

**Figure 2:**
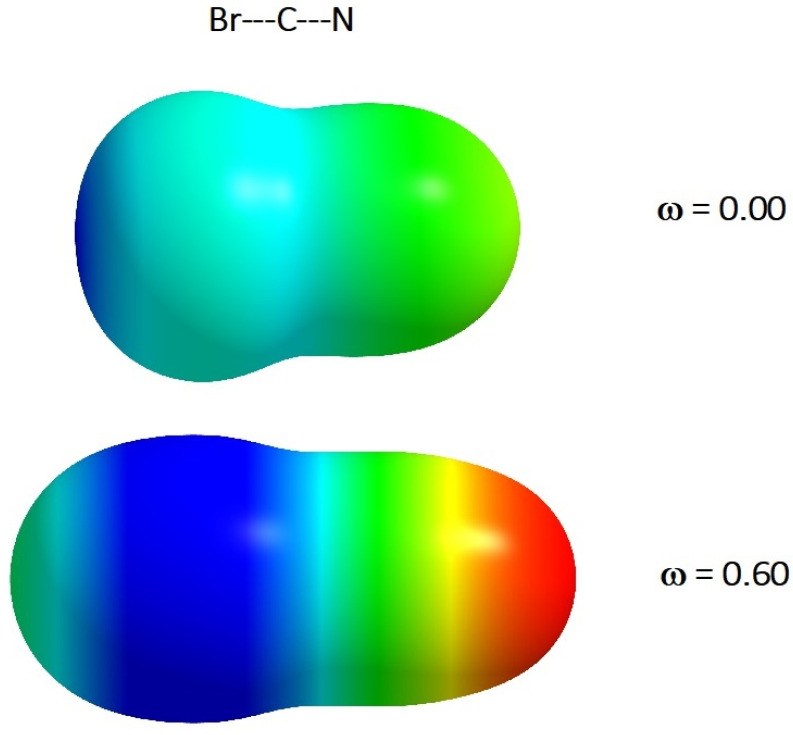
The *ω*B97X/aug-cc-pVTZ calculated MEP on the 0.001 a.u. isodensity surface of BrCN molecule (for the free molecule as well as for the molecule upon confinement). The meaning of the colors is explained in the text.

The choice of the BrCN molecule for our investigations was connected with the fact that this type of system is often discussed in the context of the possibility of forming halogen bonds (σ-hole concept based on MEP) in the molecular complexes [24,25,26]. Additionally, the simplest HF molecule was included in our consideration in order to obtain a more general conclusion. It is worth underlining that all computations of the MEP were performed for the molecular structures relaxed in the presence of cylindrical harmonic potential. This is connected with the fact that the bond distances in rigid molecules are shortened upon spatial confinement in comparison with the molecules in vacuum [23,27,28]. It has been shown that this effect strongly influences the electrical properties of molecular systems (distribution of charges, dipole moment, polarizability, etc.) [20,21,22,23,27,28,29]. MEPs have been calculated using Equation (1). It should be again noticed that the MEP at a given point around the molecule is defined as the force acting (or potential energy) on a positive test charge located at the point through the electrical charge cloud generated through the molecules’ electrons and nuclei. The MEP is often visualized through mapping its values onto the surface, reflecting the molecules’ boundaries. In our case, the molecules’ boundaries have been generated through a constant value of electron density (0.001 a.u.). The AIMAll program was used to process the data and generate the MEP onto the molecular surface [30]. It should be noticed that in our visualization, the negative MEP (red, green and yellow colors) corresponds to a situation when the positive test charge is attracted by electron density (see Figure 1 and Figure 2). On the other hand, the blue color represents positive MEP. In this region, a proton is repelled by the atomic nuclei (there is low electron density in this region). The intensity of the colors corresponds to the absolute value of MEP (the most negative value is indicated by red).

The results of the calculations of MEP (in the form of electrostatic potential maps) for the investigated molecules (for *ω* = 0 and *ω* = 0.6) are presented in Figure 1 and Figure 2.

**Figure 3:**
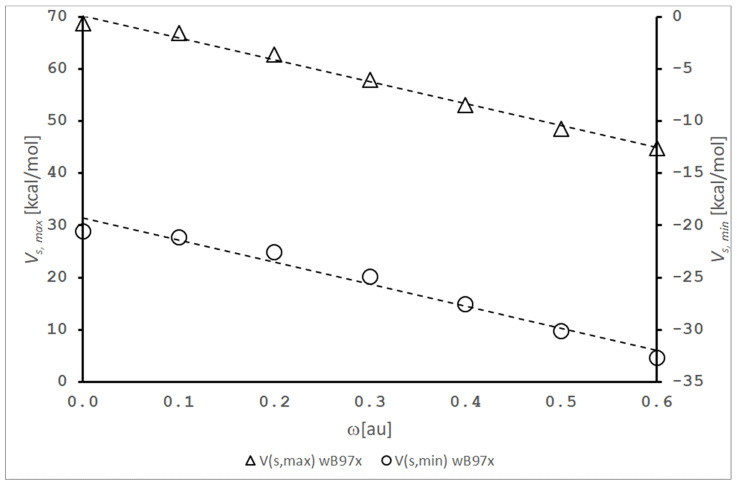
The *V_s,max_* (the maximum values of MEP) and *V_s,min_* (the minimum values of MEP) as a function of the strength confinement (*ω*) for the HF molecule. R^2^ is equal to 0.9912 and 0.9712 for *V_s,max_* and *V_s,min_*, respectively.

A short inspection of the plots presented in these figures allows us to conclude that the spatial restriction significantly changes in the MEP in two aspects. Firstly, we can observe the change in shape of the MEP of both the HF and BrCN molecules. In particular, it is clearly visible in the case of the BrCN molecule. The shape is more extended in axial direction and simultaneously becomes thinner in the equatorial direction. Due to the cylindrical symmetry of the confining potential, this result is not unexpected. The dependence of the values of MEP around the space of the molecules is more significant and complex. The results depicted in Figure 1 and Figure 2 also demonstrate how the cylindrical harmonic confining potential influences the location of the maximum (*V_s_,_max_*) and minimum (*V_s,min_*) values of MEP. It should be noticed that *V_s_,_max_* and *V_s_,_min_* correspond to the positive and negative of the MEP of the investigated molecules, respectively. In Figure 1, we cannot precisely observe that the value of *V_s,max_* of the confined HF molecule significantly decreases in comparison with the situation without spatial restriction. This finding becomes clear after analyzing the results presented in Figure 3. Here, the decrease in the values of *V_s_,_max_* (from $V_{s,max}^{\omega =0}=68.7\;\frac{\mathrm{kcal}}{\mathrm{mol}}$ to $V_{s,max}^{\omega =0.6}=44.9\;\frac{\mathrm{kcal}}{\mathrm{mol}}$) upon the increase in confinement strength is clearly observed and this relation is almost linear. It should be noticed that *V_s_,_max_* is always located on the extension of the covalent bond H-F (left side of the H atom, Figure 1). On the other hand, *V_s,min_* becomes more negative in the same conditions (from $V_{s,min}^{\omega =0}=-20.6\;\frac{\mathrm{kcal}}{\mathrm{mol}}$ to $V_{s,min}^{\omega =0.6}=-32.7\;\frac{\mathrm{kcal}}{\mathrm{mol}}$). However, in this case, we observe significant qualitative changing connected with the location of *V_s,min_*_._ For the free HF molecule, $V_{s,min}^{\omega =0}$ is located around the F atom, but for the potential strength (*ω ≈* 0.3), *V_s,min_* is located on the extension of the covalent bond H-F (right side of the F atom, Figure 1).

More drastic changes are observed for the BrCN molecule. The significant reorganization of the MEP is observed in the presence of spatial restriction in comparison with the free molecule ($V_{s,max}^{\omega =0}=43.9\;\frac{\mathrm{kcal}}{\mathrm{mol}}$ and $V_{s,min}^{\omega =0}=-31.9\;\frac{\mathrm{kcal}}{\mathrm{mol}}$). For the strongest spatial confinement considered here, $V_{s,max}^{\omega =0.6}$ is equal to 56.6 $\frac{\mathrm{kcal}}{\mathrm{mol}}$ and is located from the side of molecule (lateral blue belt in Figure 2) opposite to the free molecule, where the maximum (and also minimum) value of the MEP is located on the opposite sites of the BrCN molecule, being an extension of the covalent bond. However, this phenomenon (i.e., the change in location of *V_s_,_max_*) is already observed for *ω ≈* 0.3. It is an important finding that may have very serious consequences in the theoretical description of molecular complexes, where the σ-hole idea is applied (see Introduction). Because the σ-hole, in the classical meaning, disappears under strong spatial confinement (in this condition (*ω* = 0.6), the value of MEP on the extension of the covalent bond Br-CN is negative and equal to −2.6 $\frac{\mathrm{kcal}}{\mathrm{mol}}$), our results suggest that the BrCN molecules may significantly lose their ability to form stable and linear halogen bonds in such conditions. The same effects are expected for similar classes of compounds (FCN, ClCN and ICN). This finding is subtly supported by our results presented in Ref. [18]. However, this conclusion must be confirmed in additional studies. The other point that is worth noting is connected with the behavior of *V_s,min_*. The location of *V_s,min_* for BrCN is not changed but their value is more negative, i.e., $V_{s,min}^{\omega =0}=-31.9\;\frac{\mathrm{kcal}}{\mathrm{mol}}$ and $V_{s,min}^{\omega =0.6}=-73.9\;\frac{\mathrm{kcal}}{\mathrm{mol}}$ (red area in Figure 2). This section may be divided by subheadings. It should provide a concise and precise description of the experimental results, their interpretation, as well as the experimental conclusions that can be drawn.

## 3. Materials and Methods

In this work, all calculations were performed using density functional theory (DFT). In particular, the semi-empirical, range-separated hybrid functional ωB97X as an approximation of the exchange–correlation energy functional in DFT [31] combined with the aug-cc-pVTZ basis set [32] were employed in our theoretical considerations. The numerical results were obtained using the Gaussian 16 Rev. C01 (Gaussian, Inc.: Wallingford, CT, USA) package [33]. In order to render the impact of the spatial restriction (orbital compression) on MEP of investigated molecules, a two-dimensional harmonic oscillator potential mimicking a cylindrical confinement was applied. Hence, the effect of orbital compression was modeled by the one-electron operator in the form [17,18,20,22,23,27,29]:

(2) $$V\left(r_{i}\right)=\frac{1}{2}\omega^{2}r_{i}^{2}=\frac{1}{2}\omega^{2}\left(x_{i}^{2}+y_{i}^{2}\right)$$

added to the Hamiltonian of a free molecule. This type of model confining potential allows us to mimic a smoothly varying potential. The *ω*, which is related to the quadratic force constant of the applied harmonic oscillator potential, defines the strength of orbital compression. The physical interpretation of the *ω* value in the context of the theory of intermolecular interactions was discussed in the previous work [20]. It is worth pointing out that the analytical potential employed herein describes solely the pure spatial confinement effect, which is directly related to the valence repulsion. This type of interaction is a result of the Pauli exclusion principle and increases rapidly when the wave functions of guest and host molecules start to overlap. It leads to orbital compression (deformation of the electron density). Hence, the analyzed models should qualitatively render the effect of confinement on the guest molecule due to the nonpolarizable, electronically inert (hard) environment. The ω values considered in this work vary from 0 to 0.6 au. In all calculations, it was assumed that the principal axis of the cylindrical harmonic oscillator potential overlaps with the molecular axis of HF and BrCN, taken to be the *z*-axis. In addition, the center of confining potential was chosen to coincide with the center of mass of the molecules. The cylindrical symmetry of the repulsive potential ensures that there is no net interaction between the confining potential and the nuclei. The structures of HF and BrCN were fully optimized in vacuum as well as in the presence of harmonic confining potential at the *ω*B97X/aug-cc-pVTZ level of theory. The MEP values obtained for a given ω were calculated based on molecular structures that had been optimized at exactly the same confinement strength.

## 4. Conclusions

In summary, the present study focuses on the investigation of behavior of the MEP as a function of the strength of spatial restriction (orbital compression). In order to model this effect, the cylindrical symmetry of the repulsive potential (two-dimensional harmonic oscillator) was applied. Our preliminary studies showed that that the spatial restriction may significantly change the picture of the MEP of molecules in a quantitative and qualitative sense. This finding suggests that the many processes that occur in such conditions (in which electrostatic interactions play important role) may show different faces in comparison with the ambient conditions. It would be interesting to propose experimental measurements for the confirmation our findings. It possible to imagine that the electron densities (rather than MEP) can be derived from the X-ray diffraction experiment, as the molecular crystals contain investigated molecules. However, the measurements should be carried out as a function of the external pressure. The model confining potential often correctly described high pressure effects. It should be noted that the two-dimensional harmonic oscillator model is a hypothetical approximation. It is mainly connected with the fact that the presented model describes only repulsive forces (this type of interaction is a result of the Pauli exclusion principle and increases rapidly when the wave functions of guest and host molecules start to overlap) but neglects the van der Waals force of attraction from the neighboring molecules. Thus, this simple model of the spatial confinement corresponds to a non-polarizable, electronically inert environment. This important restriction should of course be taken into account. However, it should be underlined that this is the first study analyzing the effect of spatial confinement on MEP of molecular systems. The authors would like to express their hope that more studies will follow so that it will be possible to generalize the conclusions presented in this work.

## Data Availability

Not applicable.

## Sample Availability

Samples of the compounds are not available from the authors.
